# Editorial: Plant resistance to soil-borne diseases

**DOI:** 10.3389/fpls.2024.1369706

**Published:** 2024-02-22

**Authors:** Xiang Tao, Wenwu Ye, Ramesh Raju Vetukuri, Sophie De Vries, Lingan Kong, Meixiang Zhang

**Affiliations:** ^1^Key Laboratory of Land Resources Evaluation and Monitoring in Southwest, Ministry of Education, College of Life Sciences, Sichuan Normal University, Chengdu, China; ^2^College of Plant Protection, Nanjing Agricultural University, Nanjing, China; ^3^Department of Plant Breeding, Swedish University of Agricultural Sciences, Alnarp, Sweden; ^4^Department of Applied Bioinformatics, Institute for Microbiology and Genetics, University of Goettingen, Goettingen, Germany; ^5^State Key Laboratory for Biology of Plant Diseases and Insect Pests, Institute of Plant Protection, Chinese Academy of Agricultural Sciences, Beijing, China; ^6^College of Life Sciences, Shaanxi Normal University, Xi’an, China

**Keywords:** soil-borne diseases, pathogenesis, genetic basis, resistant genes, biocontrol

Soil-borne plant diseases, caused by fungi, oomycetes, bacteria, viruses, or nematodes dwelling in the soil, continue to pose substantial challenges to global agriculture, impacting crop yield and sustainability. These pathogens use plants as hosts for their development and soil as an appropriate environmental condition for both their spread and long-term survival. Plant hosts have evolved powerful immune strategies to defend against pathogens. To counteract this, phytopathogens have evolved intricate virulence mechanisms to evade detection by the host and/or to suppress these immune responses. A deep understanding of the mechanisms of soil-borne disease resistance is crucial for developing disease control technologies.

In recent years, significant progress has been made in understanding the complexity of these diseases and in developing innovative management strategies. We organized this Research Topic on “*Plant Resistance to Soil-Borne Diseases*” with a primary focus on the genetic basis of plant resistance and effective strategies against soil-borne plant diseases. This Research Topic is comprised of seven articles, which cover tobacco target spot disease (Tang et al.), soil-borne diseases induced by autotoxins (Chen et al.), sunflower basal stalk rot (Talukder et al.), soybean brown stem rot (McCabe et al.), *Phytophthora*-associated plant diseases (Lin et al.), and charcoal rot disease (Shirai and Eulgem, Degani et al.). Based on the research content and perspective, we have broadly categorized these articles into the following three themes: (i) pathogenesis of soil-borne plant diseases, (ii) identification of key genes involved in plant resistance to soil-borne diseases, and (iii) biological control of plant soil-borne diseases.

## Pathogenesis of soil-borne plant diseases

In this Research Topic, three aspects are investigated and discussed with respect to the pathogenesis of soil-borne diseases: autotoxins in plants, tobacco target spot disease, and a minireview on the global pathogen *Macrophomina phaseolina.* Autotoxins are a class of metabolites generated by plant roots, stems, leaves, and fruits, constituting the largest inputs of chemical substances into the rhizosphere. Continuous tobacco cropping poses challenges, leading to poor growth, a significant yield decrease, and an increased incidence of soil-borne diseases due to the accumulation of autotoxins in rhizosphere soil. Chen et al. summarized the types and composition of tobacco autotoxins under continuous cropping systems and proposed a strategy for managing tobacco autotoxicity. This strategy involves combining the breeding of superior varieties with adjustments to cropping systems, the induction of plant immunity, and the optimization of cultivation and biological control measures. The article contributes valuable insights into understanding and addressing challenges associated with autotoxins in continuous tobacco cropping systems. Tobacco target spot disease is caused by the ubiquitous soil-borne phytopathogen *Rhizoctonia solani*. Tang et al. characterized the expression profiling of *R. solani* during the infection process using RNA sequencing. They identified that genes related to plant cell wall degradation, pectin metabolism, pectin catabolism, and reactive oxygen species (ROS) scavenging play critical roles during plant infection in this system. *Macrophomina phaseolina* (Tassi) Goid is a globally distributed crop destroyer responsible for a root and stem disease characterized by black specks in the host tissue, known as charcoal rot disease (CRD). This pathogen affects more than 500 plant species and can even infect humans. Shirai and Eulgem reviewed its agricultural impact, complex disease cycle, control strategies, and the molecular mechanisms of pathogen-host interaction.

## Identification of key genes involved in plant resistance to soil-borne diseases

*Sclerotinia sclerotiorum* is a serious pathogen causing severe basal stalk rot disease in cultivated sunflowers (*Helianthus annuus* L.). Talukder et al. developed an advanced backcross population (AB-QTL) through a cross between the inbred line HA 89 and a wild annual resistant sunflower, *H. petiolaris* PI 435843. Genotyping-by-sequencing and a genetic linkage map were developed, leading to the successful identification of 14 quantitative trait loci (QTL) associated with basal stalk rot resistance derived from *H. petiolaris*. These 14 QTL are located on nine chromosomes. Brown stem rot, caused by *Phialophora gregata*, can reduce soybean yields by up to 38%. Recent studies suggest the presence of three dominant and independent resistant genes, *Rbs1*, *Rbs2*, and *Rbs3*, within the same 41 kb interval in the historical *Rbs* loci. McCabe et al. developed virus-induced gene silencing (VIGS) constructs to target the five gene clusters in the *Rbs* locus. Among them, silencing of the B1a and B2 clusters resulted in a complete loss of *Rbs1*-mediated resistance to *P. gregata*, but was unable to silence *P. gregata* resistance in either PI 437833 (*Rbs2*) or PI 437970 (*Rbs3*). These findings support the oligogenic inheritance model proposed by Bachman and Nickell and suggest that this region on chromosome 16 contains additional *P. gregata* resistance genes.

## Biological control of plant soil-borne diseases

The oomycete pathogen *Phytophthora* causes devastating diseases in diverse important agricultural crops. Lin et al. provided abundant evidence demonstrating that *Lysobacter enzymogenes* effectively protects host plants from various *Phytophthora* diseases. The *L. enzymogenes* OH11 demonstrated inhibition of mycelial growth, digestion of cysts, suppression of cyst germination, and elicitation of plant immune responses. Furthermore, they found that *L. enzymogenes* prevents *Phytophthora* infection via multiple previously unknown mechanisms. These results highlighted its potential as a biocontrol agent. Degani et al. explored the efficacy of five *Trichoderma* species and Azoxystrobin (AS), an already field-validated treatment, applied separately or in combination to treat cotton seedlings, with the aim of reducing cotton CRD. They present cotton growers worldwide with an improved control protocol to minimize the damages caused by CRD.

## Author contributions

XT: Writing – original draft, Writing – review & editing. WY: Writing – original draft, Writing – review & editing. RV: Writing – original draft, Writing – review & editing. SD: Writing – original draft, Writing – review & editing. LK: Writing – original draft, Writing – review & editing. MZ: Writing – original draft, Writing – review & editing.

